# Feasibility of gray-blood late gadolinium enhancement evaluation in young patients with congenital and acquired heart disease

**DOI:** 10.3389/fcvm.2023.1269412

**Published:** 2023-10-17

**Authors:** Cesar Gonzalez de Alba, Mehdi H. Moghari, Lorna P. Browne, Richard M. Friesen, Brian Fonseca, LaDonna J. Malone

**Affiliations:** ^1^ Division of Cardiology, Heart Institute, Children’s Hospital Colorado, University of Colorado, Aurora, CO, United States; ^2^ Department of Radiology, University of Colorado, Aurora, CO, United States; ^3^ Department of Radiology, Children’s Hospital Colorado, Aurora, CO, United States

**Keywords:** cardiovascular magnetic resonance, congenital heart disease, late gadolinium enhancement, myocardial fibrosis, phase-sensitive inversion recovery, subendocardial fibrosis

## Abstract

**Background:**

Late gadolinium enhancement (LGE) sequences have become common in pediatric cardiovascular magnetic resonance (CMR) to assess for myocardial fibrosis. Bright-blood late gadolinium enhancement (BB-LGE) by conventional phase-sensitive inversion recovery (PSIR) is commonly utilized, but similar inversion time (TI) value of fibrosis and left ventricular (LV) blood pool can make subendocardial areas difficult to assess. A gray-blood LGE (GB-LGE) technique has been described, targeting nulling of the LV blood pool and demonstrating improvement in ischemic scar detection over BB-LGE in adult patients. We sought to evaluate the feasibility of the GB-LGE technique in a young population with congenital and acquired heart disease and compare its ability to detect subendocardial scar to conventional BB-LGE.

**Methods:**

Seventy-six consecutive patients referred for clinical CMR underwent both BB-LGE and GB-LGE on 1.5 T and 3 T scanners. Conventional PSIR sequences were obtained with TI to null the myocardium (BB-LGE) in short-axis and horizontal long-axis stacks. Same PSIR stacks were immediately repeated with TI to null the blood pool (GB-LGE). Both sequences were reviewed separately a week apart by two readers, blinded to the initial clinical interpretation. Studies were analyzed for overall image quality, confidence in scar detection, confidence in detection of LGE, LGE class, inter- and intra-observer agreement for the presence of scar, and intraclass correlation coefficient (ICC) for total scar burden.

**Results:**

Overall confidence in myocardial scar detection by BB-LGE or GB-LGE as well as grading of image quality were not statistically different [(*p* = 1 and *p* = 1) and (*p* = 0.53, *p* = 0.18), respectively]. There was very good inter-observer agreement for the presence of scar on BB-LGE (*K* = 0.88, 95% CI 0.77–0.99) and GB-LGE (*K* = 0.84, 95% CI 0.7–0.96), as well as excellent intra-observer agreement for both readers (*K* = 0.93, 95% CI 0.87–0.99; and *K* = 0.81, 95% CI 0.69–0.95). Interclass correlation coefficient for total scar burden was excellent for BB-LGE (ICC = 0.98, 95% CI 0.96–0.99) and GB-LGE (ICC = 0.94, 95% CI 0.91–0.97).

**Conclusions:**

The GB-LGE technique is feasible in the pediatric population with congenital and acquired heart disease. It can detect subendocardial/ischemic scar similar to conventional bright-blood PSIR sequences in the pediatric population.

## Introduction

Late gadolinium enhancement (LGE) sequences have become commonly utilized in pediatric cardiovascular magnetic resonance (CMR) exams to assess for myocardial scar/fibrosis. The presence of LGE in various pediatric acquired and congenital heart diseases is well documented, and its distribution patterns can have important prognostic implications (1–6).

Phase-sensitive inversion recovery (PSIR) is a commonly utilized CMR sequence to evaluate for LGE. An inversion time (TI) is chosen, typically in mid to late diastole to decrease cardiac motion, to primarily null the myocardial signal. This technique is also referred to as bright-blood gadolinium enhancement (BB-LGE) as it leaves a relatively bright-blood pool and a dark myocardium, with enhancement of areas of myocardial fibrosis, which typically carry a higher gadolinium concentration and thus a shorter TI time (7, 8). One downside of BB-LGE is that depending on the chosen TI, this sequence may show similar TI value of scar/fibrosis and the left ventricular (LV) blood pool, which may lead to difficulty in assessing areas of LGE, particularly at the subendocardial surface (9).

To address this difficulty, a gray-blood LGE (GB-LGE) technique has been described in the adult population. A shorter TI is utilized, targeting additional nulling of the LV blood pool and leaving it gray along with a dark myocardium, allowing for a better differentiation of LGE at the subendocardial surface (9, 10). To our knowledge, utilization of this technique has not been reported in the pediatric population. We set out to evaluate the feasibility of the GB-LGE technique in a young population with congenital and acquired heart disease, and compare its ability to detect subendocardial/ischemic scar to conventional BB-LGE.

## Materials and methods

### Study population

This retrospective study was carried out between June 2021 and April 2022 at the Children's Hospital of Colorado. A total of 80 consecutive patients with various types of congenital and acquired heart disease referred for further evaluation by CMR were included in this study. Inclusion criteria encompassed patients diagnosed with congenital heart disease who were referred for clinically indicated cardiovascular MR examinations. Exclusion criteria consisted of patients with claustrophobia or an inability to tolerate extended scan durations and individuals with metallic objects obstructing the visualization of the left ventricle.

Studies were performed on 1.5 T and 3 T Philips Ingenia scanners (Philips Healthcare, Best, Netherlands) per usual protocol with no strict randomization performed. Our institutional review board approved this study.

### LGE sequences

**Figure 1:**
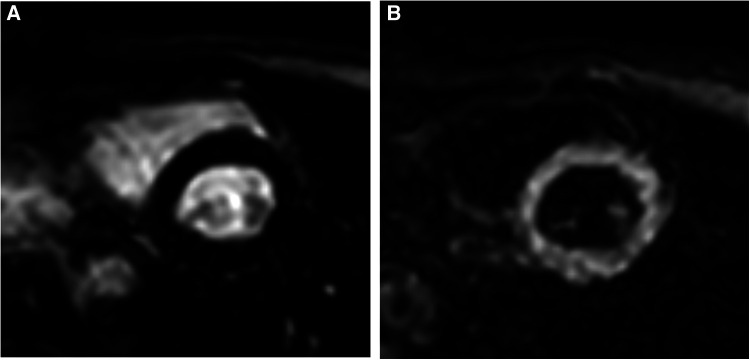
TI selection on the Look Locker sequence. (**A**) Typical inversion time utilized for conventional bright-blood LGE; blood pool is bright and myocardium is nulled. (**B**) Typical inversion time for gray-blood LGE; blood pool is nulled and myocardium is bright. TI for gray-blood LGE occurs prior to the typical TI for bright-blood LGE.

All patients underwent a routine CMR study with protocols dedicated to their specific diagnosis. Approximately 8–10 min after an intravenous injection of gadobenate (0.2 ml/kg/dose), a Look Locker sequence utilizing a single short-axis slice was used to select the appropriate TI (Figure 1) (11). Conventional BB-LGE PSIR sequences were subsequently obtained in the horizontal long-axis view (three slices) and short-axis view (8–12 slices) covering the entire LV (7). Bright-blood PSIR LGE sequences were obtained by utilizing a TI to null the myocardium. Gray-blood PSIR LGE sequences used a TI to achieve nulling of the blood pool. Identical slices for both BB-LGE PSIR and GB-LGE PSIR were utilized. Only the TI parameter was adjusted in the conventional PSIR protocols to turn the BB-LGE into GB-LGE images. All other imaging parameters for both GB-LGE and BB-LGE were the same as follows: field strength = 1.5 T and 3.0 T; field of view = 350 mm^2^ × 350 mm^2^; readout type = T_1_ turbo field echo; slice thickness = 7–8 mm; acquired spatial resolution = 1.5 mm^2^ × 1.8 mm^2^, reconstructed spatial resolution ≈ 0.9 mm^2^ × 0.9 mm^2^; repetition time ≈ 6.1 ms; echo time ≈ 3 ms; and flip angle = 25°.

### Image analysis

Two independent readers (CG, LM) with additional CMR subspecialty training, blinded to initial clinical interpretation, reviewed all BB-LGE and GB-LGE PSIR images separately, at least a week apart, in a random order. All images were analyzed using PACS Synapse 5 (FUJIFILM Medical Systems, Stamford, CT, USA).

Studies were analyzed utilizing Holtackers et al. LGE image assessment with some modifications (8). This included overall image quality rating on a 4-point Likert scale: (0) non-diagnostic, (1) low (≥ two segments non-diagnostic), (2) medium (one segment non-diagnostic), or (3) high (all segments correctly identified). Non-diagnostic images were excluded from further analysis. LGE type was classified as: (0) no scar, (1) ischemic/subendocardial scar, or (2) both ischemic/subendocardial and non-ischemic scar. For classification discrepancies between both readers, a third expert reader (BF) provided their interpretation for a final consensus. Transmural extent of all types of scars was evaluated as a percentage of local total wall thickness per segment based on the 17-segment American Heart Association model. The transmural extent on each segment was evaluated on a 5-point Likert scale: (0) no scar, (1) 1%–25%, (2) 26%–50%, (3) 51%–75%, or (4) 76%–100%. Total scar burden was then calculated as the sum of all segments multiplied by their corresponding maximum transmural percentage with a maximum possible scar burden = 17. Finally, observer confidence in scar detection analysis was rated using a binary scale: (0) not confident and (2) confident. Intra- and inter-observer agreement was performed for the presence of scar.

### Statistical analysis

All statistical analyses were performed using MedCalc Statistical Software version 20.218 (MedCalc Software bv, Ostend, Belgium; https://www.medcalc.org; 2023). Results were expressed as median with interquartile range, mean ± standard deviation, or percentage as deemed fit. Observer confidence in scar detection and overall image quality between both methods were evaluated using the McNemar test and Wilcoxon signed-rank test, respectively. Differences in subendocardial/ischemic scar detection between BB-LGE and GB-LGE sequences were evaluated using McNemar tests. Differences in total scar burden between both methods were evaluated using a paired-sample *t*-test for normally distributed data or Wilcoxon signed-rank test for non-normally distributed data. Data normality was assessed by the Shapiro–Wilk test. Intra- and inter-observer agreement for the presence of scar was evaluated by Cohen's kappa and Fleiss’ kappa coefficients, respectively. Intra- and inter-observer variability in total scar burden was evaluated by calculating an intraclass correlation coefficient (ICC) (average measure). A *K* value of <0.2 was considered poor, 0.21–0.4 fair, 0.41–0.6 moderate, 0.61–0.8 good, and 0.81–1.00 very good. Bland–Altman plots were utilized to compare total scar burden assessment by both methods for both readers. Statistical tests were two-tailed and *p*-values <0.05 were considered significant.

## Results

### Study population

Baseline patient characteristics are described in Table 1. Eighty patients were initially selected for this study. Four patients (5%) were excluded from further analysis due to missing or mismatched PSIR sets (i.e., slices did not match between sets due to patient movement necessitating replanning of the second set leading to different slice locations, and/or different patient breath hold level yielding different imaging location of the heart). One more patient in the GB-LGE group was excluded due to an incomplete short-axis stack, but their complete BB-LGE stack was kept for analysis. Statistical analysis was performed on 76 patients (95%) who had complete conventional BB-LGE sequences and 75 patients (94%) with complete GB-LGE sequences.

**Table 1:** Baseline demographics (*N* = 76).

Age at study (years)	16.3 (6)
Gender	
Female (*n*, %)	34 (45%)
Male (*n*, %)	42 (55%)
Weight (kg)	61.2 (19)
Height (cm)	155.8 (50.9)
BSA (m^2^)	1.7 (0.36)
Field strength	
1.5 T	43 (57%)
3 T	33 (43%)
Diagnoses (*n*, %)	
Cardiomyopathy	15 (20%)
Aortopathy (including bicuspid aortic valve)	15 (20%)
Myocarditis	8 (11%)
Tetralogy of Fallot	7 (9%)
Turner syndrome	5 (7%)
Single ventricle (at various palliation stages)	5 (7%)
ARVC rule-out	4 (5%)
Ischemic pathology (Kawasaki, LAD thrombus)	3 (4%)
D-transposition of the great arteries (repaired)	3 (4%)
Other diagnoses	11 (14%)

ARVC, arrhythmogenic right ventricular cardiomyopathy; LAD, left anterior descending artery; BSA, body surface area.

Numbers are expressed as median with interquartile range (IQR).

### LV scar detection

**Figure 2:**
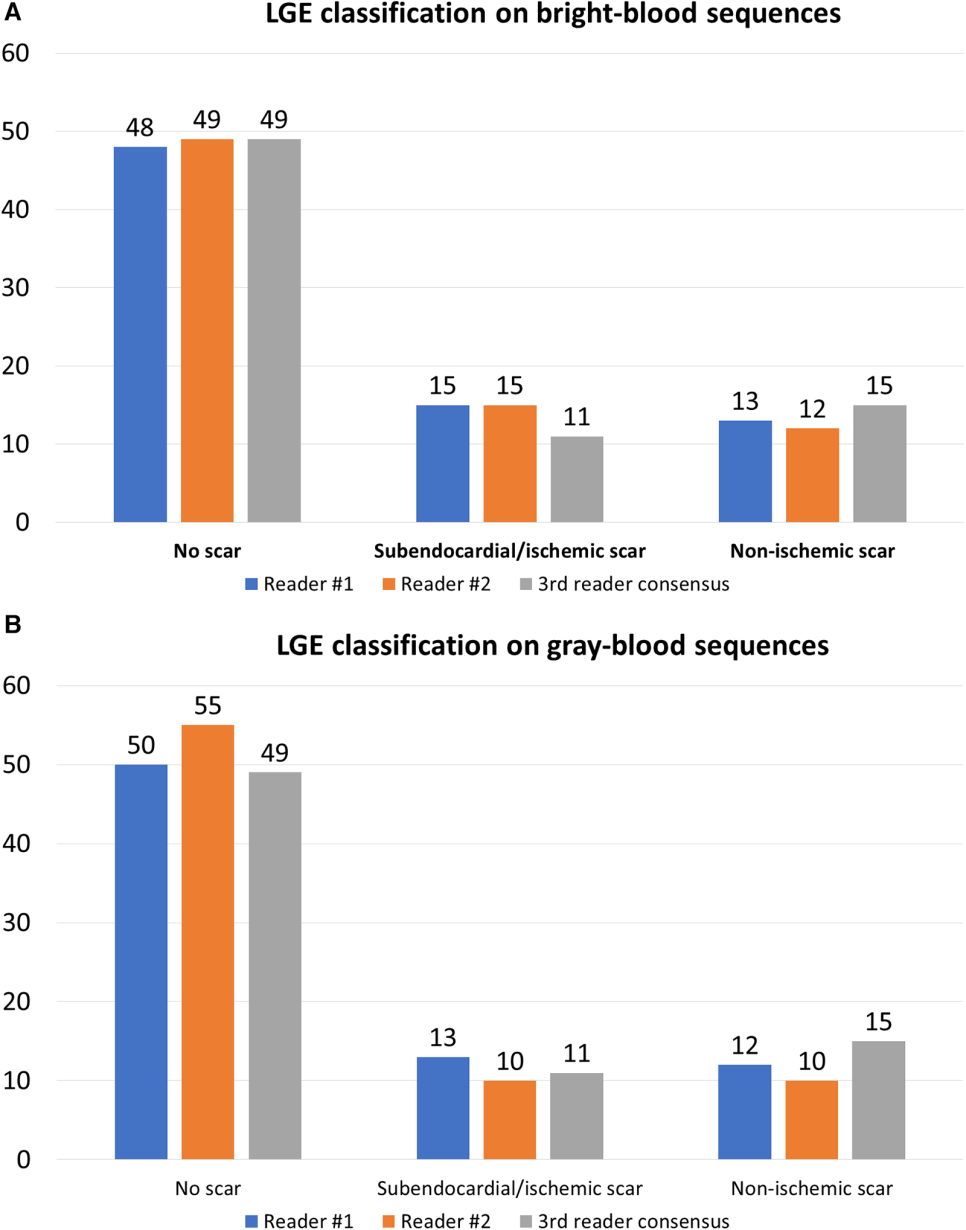
LGE classification on bright-blood (**A**) and gray-blood sequences (**B**).

Classification of detected LV scar or absence of scar is detailed in Figure 2. There was no significant difference in ischemic/subendocardial LV scar pattern detection between both techniques for reader #1 who diagnosed it in 15 subjects by BB-LGE and in 13 subjects by GB-LGE (20% vs. 17%, *p* = 0.625). However, there was a statistically significant difference for reader #2, who detected myocardial scar in 15 subjects by BB-LGE and 10 subjects by GB-LGE (21% vs. 13%, *p* = 0.0313).

**Figure 3:**
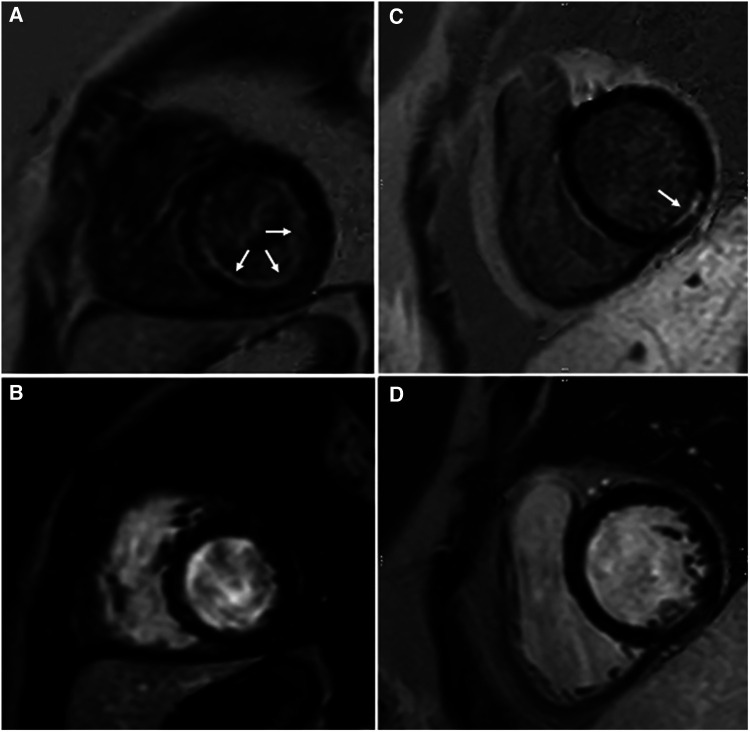
Observed subendocardial/ischemic LGE patterns. Endocardial fibroelastosis (arrows) in a patient with critical aortic stenosis is best discerned on GB-LGE (**A**) compared to BB-LGE (**B**). LGE (arrow) in a patient with coronary artery complications after Kawasaki disease is best discerned from blood pool in GB-LGE (**C**) compared to BB-LGE (**D**).

**Figure 4:**
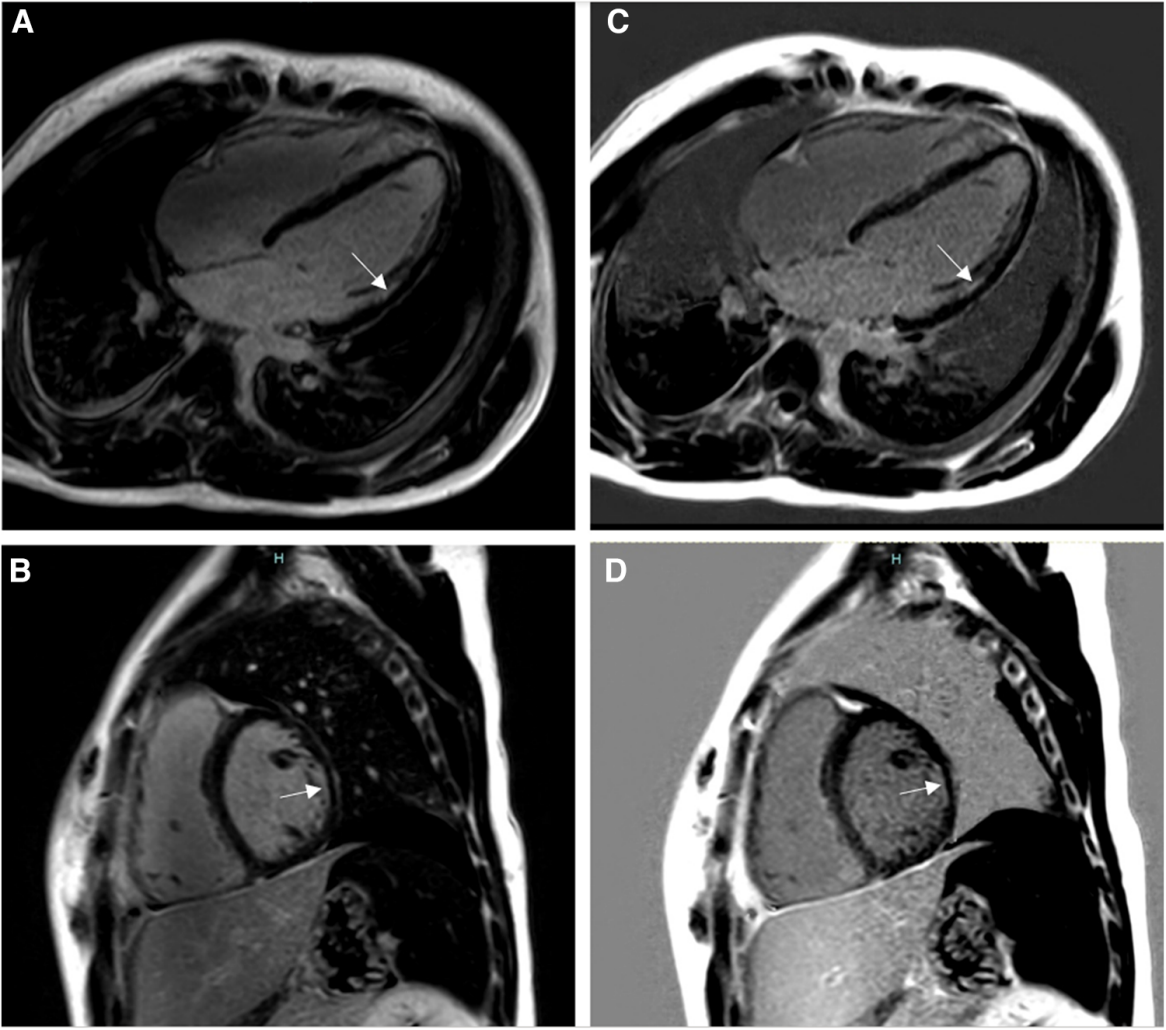
Fourteen-year-old admitted for ventricular fibrillation. (**A,B**) show conventional BB-LGE sequences; subendocardial scar is somewhat difficult to visualize given its similar signal intensity to the blood pool. (**C,D**) show gray-blood LGE sequences; the scar is relatively easier to see. Ultimately, utilization of both sequences increased the readers’ confidence in correctly diagnosing it.

Discrepancies in diagnosis of any type of scar or no scar between the two readers were re-assessed by a third reader. There were 11 subjects (15%) with a final diagnosis of subendocardial/ischemic scar, 15 subjects (20%) with non-ischemic scar, and 49 subjects (65%) with no scar. Five patients with subendocardial/ischemic scar had their study performed in a 3 T scanner, while six patients had it in a 1.5 T scanner. Diagnoses of patients with subendocardial/ischemic scar included patients with coronary artery complications after Kawasaki disease (two patients, Figure 3), aortic stenosis (two patients, Figure 3), dilated cardiomyopathy (two patients), repaired tetralogy of Fallot without known coronary artery disease (two patients), arrhythmogenic right ventricular cardiomyopathy (one patient), one patient with suspected perinatal coronary event, and one patient with history of cardiac arrest and ventricular fibrillation (Figure 4).

Compared to the third reader re-assessment, ischemic scar was identified in 10 subjects (91%) on BB-LGE and 11 subjects (100%) on GB-LGE by reader 1, while reader 2 identified it in 11 subjects (100%) on BB-LGE and 10 subjects (91%) on GB-LGE. Non-ischemic scar was identified in 11 subjects (73%) by reader 1 on both GB-LGE and BB-LGE, while reader 2 identified it on 11 subjects (73%) by BB-LGE and 10 subjects (67%) on GB-LGE. The absence of scar was identified by reader 1 on 46 subjects (94%) by BB-LGE and GB-LGE, while reader 2 identified this on 48 subjects (98%) on BB-LGE and 49 subjects (100%) on GB-LGE.

**Figure 5:**
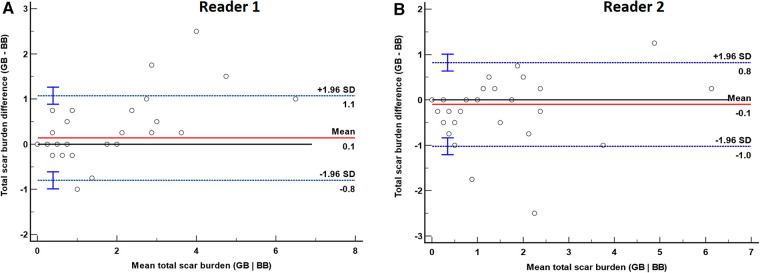
Bland–Altman plot of total scar burden evaluation by gray-blood (GB) and bright-blood (BB) sequences for readers 1 (**A**) and 2 (**B**). The red solid line represents the mean difference between methods, and the blue dashed lines are the limits of agreement (LoA) with their corresponding 95% confidence interval. (**A**) Bland–Altman plot for reader 1. There was a significant bias with a mean of 0.14 (*p* = 0.0156), LoA −0.8 and +1.1. (**B**) Bland–Altman plot for reader 2. There was no significant bias with a mean of −0.1 (*p* = 0.07), LoA −1.0 and +0.8.

Total scar burden in subjects with subendocardial/ischemic scar was statistically different between BB-LGE and GB-LGE for reader 1 (median 1.38, IQR = 1.81, vs. median 1.38, IQR = 3.31, *p* = 0.02) but not for reader 2 (median 1.3, IQR = 2.3 vs. median 0.6, IQR = 2.3, *p* = 0.28). Bland–Altman plots of total scar burden assessment by both methods were performed for both readers (Figure 5). These demonstrated a statistically significant bias for reader 1 (mean difference +0.1, *p* = 0.0156), but not for reader 2 (mean difference −0.1, *p* = 0.07).

### Overall image quality

There were no statistically significant differences in overall image quality classification between methods for both readers (*p* = 0.53 and *p* = 0.18). High image quality was observed in 58 patients (76%) with BB-LGE vs. 55 patients (72%) with GB-LGE for reader 1, and 46 patients (61%) with BB-LGE vs. 38 (51%) with GB-LGE for reader 2. Medium image quality was observed in 16 patients (21%) with BB-LGE vs. 20 patients (27%) with GB-LGE for reader 1, and 29 patients (38%) with BB-LGE vs. 36 patients (48%) with GB-LGE for reader 2. Low-quality images were observed in two patients (3%) with BB-LGE vs. one patient (1%) with GB-LGE for reader 1, and in one patient (1%) for both BB-LGE and GB-LGE for reader 2. There were no non-diagnostic images for both readers.

### Observer confidence in scar detection

There were no significant differences in observer confidence to detect myocardial scar between sequences for both readers. There was overall excellent confidence in reading both conventional BB-LGE sequences (99%) and GB-LGE (100%) for reader 1 as well as for reader 2 (99% for both methods). From those with complete BB-LGE and GB-LGE sets, only five patients (7%) had a GB-LGE sequence performed first, while all others (93%) had conventional BB-LGE performed initially. Of those five patients, two had subendocardial/ischemic scar that was correctly identified by both readers on both methods, one patient had non-ischemic scar that was detected by GB-LGE by reader 1 but was not detected on any sequence by reader 2, and two patients had no scar which was correctly diagnosed by both readers on both sequences.

### Intra- and inter-observer agreement

There was a very good intra-observer agreement between sequences for both readers (*K* = 0.93 and *K* = 0.81), as well as very good inter-observer variability between readers (*K* = 0.88 and *K* = 0.84) in the overall assessment for the presence of scar. The intraclass correlation coefficient for total scar burden assessment had excellent intra-observer agreement between BB-LGE and GB-LGE for both readers (both readers ICC = 0.96). The inter-observer variability in total scar burden detection was also excellent between readers for conventional BB-LGE (ICC = 0.98) and GB-LGE (ICC = 0.94).

## Discussion

Our study aimed to assess the feasibility of gray-blood LGE sequences and compare it to conventional BB-LGE, particularly to detect subendocardial/ischemic scarring in a pediatric population with congenital and acquired heart disease. We did not find any significant differences in image quality (*p* = 0.53 and *p* = 0.18), overall confidence in scar detection, and detection of non-ischemic scar or absence of scar between BB-LGE and GB-LGE sequences for both readers. We also found no non-diagnostic images for both sequences, and only one to two patients with low-quality images in both sequences. There were no significant differences in detection of non-ischemic scar or absence of scar between methods for both readers.

There were mixed results between our two readers in subendocardial/ischemic LV scar detection, with one finding a statistically significant difference between methods (*p* = 0.0313) but not the other (*p* = 0.625). Mixed results were also found for total scar burden assessment, with a significant difference found by one of our readers (*p* = 0.02) but not by the other (*p* = 0.28). Despite this, we still found very good inter-observer (*K* = 0.88 and *K* = 0.84) and intra-observer (*K* = 0.93 and *K* = 0.81) agreement in assessment of scar between sequences. There was also an excellent intraclass correlation coefficient (both readers ICC = 0.96) in total scar burden assessment between sequences and readers. Finally, while Bland–Altman plots of total scar burden assessment by both methods for both readers demonstrated a statistically significant bias for reader 1 (mean difference +0.1, *p* = 0.0156), but not for reader 2 (mean difference −0.1, *p* = 0.07), a mean average discrepancy of +0.1 or −0.1, seen from a clinical perspective, likely points to a relative equivalence between both methods.

We utilized a third CMR reader to assess the discrepancies between readers and give consensus for a final diagnosis of subendocardial/ischemic scar. There were a total of 11 patients (15%) with a final diagnosis of subendocardial/ischemic scar and 15 patients (20%) with a final diagnosis of non-ischemic scar. When compared to the third reader's assessment, readers and 2 identified four more cases of subendocardial scar on BB-LGE (15 patients for both readers vs. 11 patients by the third reader); however, these discrepancies decreased on GB-LGE (13 patients for reader 1 and 10 patients for reader 2). Regarding non-ischemic scar detection, both readers detected less cases compared to the third reader on both sequences; reader 1 detected two less cases on BB-LGE and three less cases on GB-LGE, while reader 2 detected three less cases on BB-LGE and five less cases on GB-LGE. Given that our numbers of mid-myocardial scar were low overall, it is difficult to establish definitive inferiority of GB-LGE in detecting this type of scar. However, given these findings and our study's limitations, our institutional practice remains to keep the gold standard BB-LGE as our routine PSIR sequence, with the use of GB-LGE only for cases where there is suspicion for subendocardial fibrosis (as an addition to BB-LGE).

Evaluation for the presence and extension of myocardial scar by CMR has become an important clinical piece in the evaluation of patients with congenital and acquired heart disease. The detection of LGE can carry both important diagnostic and prognostic implications. In various types of cardiomyopathies, the presence of LGE as well and the number of involved LV segments correlate with worse global LV systolic function, increased risk for sudden cardiac death, and ventricular arrhythmia (12, 13). LGE is also an independent predictor of ventricular arrhythmias and death in Duchenne's muscular dystrophy (14). In severe LV outflow obstructive pathologies, a diffuse fibrous lining of the endocardium known as endocardial fibroelastosis (EFE) can be seen as LGE in CMR, carrying a poor prognosis in many cases (15). LGE enhancement has also been associated with adverse outcomes in patients with repaired tetralogy of Fallot (16, 17).

Conventional PSIR using BB-LGE sequences are commonly utilized to evaluate for LGE in patients with suspected ischemic or non-ischemic myocardial scar (18–22). However, differentiation of scar at the subendocardial level may be difficult in cases where the blood pool remains too bright, which may lead to missing the detection of subendocardial/ischemic scar. Delayed viability sequences have been described to improve the assessment for subendocardial scar in pediatric patients (15). However, obtaining these sequences require a waiting time of >25 min after gadolinium contrast injection, whereas GB-LGE sequences can be obtained after 8–10 min post-contrast injection. Holtackers et al. found a statistically significant difference in ischemic scar diagnosis by dark-blood LGE vs. conventional BB-LGE (97 vs. 98, *p* = 0.008) (10). Our study findings, however, were not as significant as those found by this group. This may be due to an overall lower patient population in our study, and especially a lower incidence of subendocardial/ischemic scar, which is inherent to studying a pediatric population where ischemic pathologies are much less likely to occur.

## Limitations

Our study had several limitations. Our patient population and sample size limited the number of patients with subendocardial/ischemic scar. In the majority of cases, BB-LGE was performed first, with GB-LGE performed first in only five cases, which may lead to a confounding factor of time after gadolinium contrast administration. Strict randomization between BB-LGE and GB-LGE was not possible in our study because preference was often given to complete our standardized protocols (which utilize BB-LGE as the standard of care) in case patients would become exhausted from the extra breath-holds, which commonly occurs in pediatric patients. Regardless, image quality and reader confidence were similar for both sequences. Finally, we did not have any histological confirmation of scar in any of our patients. However, there has been histopathological validation of GB-LGE without additional magnetization preparation in a porcine animal model, which demonstrated superior visualization and quantification of scar compared to bright-blood sequences (23).

## Conclusions

Gray-blood late gadolinium enhancement technique is feasible in the pediatric population with congenital and acquired heart disease. It can detect subendocardial/ischemic scar similar to conventional bright-blood PSIR sequences. Further studies in larger pediatric populations, including BB-LGE and GB-LGE randomization, may be needed to demonstrate a larger effect as demonstrated by other studies.

## Data Availability

The raw data supporting the conclusions of this article will be made available by the authors, without undue reservation.
